# Changes in the expression of the type 2 diabetes-associated gene *VPS13C* in the β-cell are associated with glucose intolerance in humans and mice

**DOI:** 10.1152/ajpendo.00074.2016

**Published:** 2016-06-21

**Authors:** Zenobia B. Mehta, Nicholas Fine, Timothy J. Pullen, Matthew C. Cane, Ming Hu, Pauline Chabosseau, Gargi Meur, Antonio Velayos-Baeza, Anthony P. Monaco, Lorella Marselli, Piero Marchetti, Guy A. Rutter

**Affiliations:** ^1^Section of Cell Biology and Functional Genomics, Imperial College London, London, United Kingdom;; ^2^Wellcome Trust Centre for Human Genetics, Oxford, United Kingdom; and; ^3^Department of Clinical and Experimental Medicine, University of Pisa, Pisa, Italy

**Keywords:** VPS13C, C2CD4A, GWAS, type 2 diabetes, β-cell

## Abstract

Single nucleotide polymorphisms (SNPs) close to the VPS13C, C2CD4A and C2CD4B genes on chromosome 15q are associated with impaired fasting glucose and increased risk of type 2 diabetes. eQTL analysis revealed an association between possession of risk (C) alleles at a previously implicated causal SNP, rs7163757, and lowered VPS13C and C2CD4A levels in islets from female (*n* = 40, *P* < 0.041) but not from male subjects. Explored using promoter-reporter assays in β-cells and other cell lines, the risk variant at rs7163757 lowered enhancer activity. Mice deleted for *Vps13c* selectively in the β-cell were generated by crossing animals bearing a floxed allele at exon 1 to mice expressing Cre recombinase under Ins1 promoter control (Ins1Cre). Whereas Vps13c^fl/fl^:Ins1Cre (βVps13cKO) mice displayed normal weight gain compared with control littermates, deletion of *Vps13c* had little effect on glucose tolerance. Pancreatic histology revealed no significant change in β-cell mass in KO mice vs. controls, and glucose-stimulated insulin secretion from isolated islets was not altered in vitro between control and βVps13cKO mice. However, a tendency was observed in female null mice for lower insulin levels and β-cell function (HOMA-B) in vivo. Furthermore, glucose-stimulated increases in intracellular free Ca^2+^ were significantly increased in islets from female KO mice, suggesting impaired Ca^2+^ sensitivity of the secretory machinery. The present data thus provide evidence for a limited role for changes in VPS13C expression in conferring altered disease risk at this locus, particularly in females, and suggest that C2CD4A may also be involved.

the incidence of type 2 diabetes (T2D) is now reaching epidemic proportions across the globe, with deaths from the disease reaching 5.1 million and disease complications costing USD 548 billion in 2013 (30). These values are expected to continue to increase, with predictions of a further 205 million sufferers by 2035 (30). T2D is a complex metabolic disease involving hyperglycemia and dyslipidemia, which together conspire to cause serious secondary macro- and microvascular complications including cardiovascular disease, retinopathy, and neuropathy (11, 19). Although it is accepted that a loss of an appropriate balance between functioning pancreatic β-cell mass and insulin action in peripheral tissues leads to abnormal glucose homeostasis, the molecular basis of T2D onset and progression is still poorly understood (31, 57).

While environmental factors such as increasingly sedentary lifestyles and obesogenic diets have a substantial impact, genetic susceptibility also plays a significant role in T2D risk (57). Correspondingly, genome-wide association studies (GWAS) have identified ∼90 loci harboring single nucleotide polymorphisms (SNPs) that confer increased disease risk (13, 21, 23, 40, 57, 65, 86). Such studies have thus led to the discovery of novel genes involved in T2D, such as T cell factor 7-like 2 (TCF7L2) (21) and SLC30A8 (58, 65). Of note, the majority of the GWAS-identified loci affect insulin secretion rather than action, further emphasising the likely role in disease etiology of impaired insulin production.

The VPS13C/C2CD4A/C2CD4B locus was first associated with T2D and glycemic traits in GWAS published in 2010 (6, 15, 22, 29, 60). Subsequent studies identified further SNPs at this genomic location associated with poorer glycemic control and T2D (12, 67, 78). The above studies encompassed a range of distinct populations and age groups, thus providing confidence that SNPs in this locus, acting via either nearby or more remotely located genes, alter genetic susceptibility to T2D. SNPs within the VPS13C/C2CD4A/B locus have been linked to a range of glycemic parameters including higher fasting proinsulin (29, 67), higher 2-h glucose and lower 2-h insulin (60, 67), as well as increased fasting glucose (15, 22, 67) and increased waist circumference (22). Two studies also associated risk alleles with lower glucose-stimulated insulin secretion (GSIS) (6, 22, 67) and others with T2D (12, 67, 83). The “lead” (GWAS index) SNP in this locus, rs7172432, is in LD with a “functional” SNP, rs7163757, previously implicated by fine mapping as the most strongly associated (*P* = 3 × 10^−19^) SNP at this locus (61, 66). rs7163757 is located in an islet stretch enhancer (50, 61, 66), again suggesting that the disease-associated SNP acts on the expression of an effector gene(s) to alter diabetes risk.

The first identified member of the highly conserved VPS13 (vacuolar protein sorting 13) family of proteins was Soi1 (or Vps13) in *Saccharomyces cerevisiae*, where it plays an important role in membrane protein trafficking between the trans-Golgi network (TGN) and the prevacuolar compartment (7). Specifically, Vps13 is involved in trafficking the protease Kex2p, a protein involved in intracellular insulin processing after overexpression of the latter in yeast (85). Subsequently, a role for this protein was demonstrated in prospore formation in *S. cerevisiae* through the regulation of phosphatidylinositol 4-phosphate [PI(4)P] generation and membrane-bending activity (48, 49).

In both humans and mice, the VPS13 family comprises four members (A–D), with VPS13A and VPS13C showing the most similarity to the yeast homolog (73). All four proteins are large and have potential functions in membrane protein trafficking, Golgi structure, and/or phosphatidylinositol metabolism (37, 47, 53, 62, 63, 73). Mutations in VPS13A and VPS13B cause the genetic diseases chorea-acanthocytosis (ChAc) and Cohen syndrome, respectively (32, 53, 71), and a loss of VPS13C function has recently been linked to early-onset Parkinson's disease (35).

VPS13C is ubiquitously expressed in mammals, with particularly high levels in pancreatic islets and β-cells (60, 67). The observations above have thus led us to hypothesize that VPS13C may play a role in the intracellular trafficking of insulin or other aspects of pancreatic β-cell function. To explore this possibility, we first determined the relationship between the possession of T2D risk alleles in humans and the expression of VPS13C, C2CD4A (C2 calcium-dependent domain 4A), and C2CD4B in human islets. Subsequently, we developed mice inactivated for Vps13c highly selectively in the β-cell by using the recently developed Ins1Cre deleter strain (33, 69). The latter is a knock-in model that avoids the complications associated with earlier insulin 2 promoter-dependent Cre's including recombination in the brain (77) and coexpression of human growth hormone (8). This approach reveals roles for Vps13c in the control of whole body glucose homeostasis, insulin secretion in vivo, and glucose-induced Ca^2+^ signal generation in the β-cell but suggests that C2CD4A may also contribute to disease risk.

## MATERIALS AND METHODS

### 

#### Materials.

All general chemicals and materials were purchased from Sigma (Dorset, UK) or Fisher Scientific (Loughborough, UK) unless otherwise indicated.

#### Generation of VPS13C antibodies.

A custom polyclonal antibody against human VPS13C, based on amino acids 1582–1882 of human VPS13C isoform 2A (UniProtKB Q709C8-1; 84% identities, 92% positives with mouse VPS13C protein Q8BX70-1, positions 1580–1879) was raised in rabbits, as recently described (84).

#### Ethics.

All in vivo procedures were conducted in accordance with UK Home Office regulations [Animals (Scientific Procedures) Act of 1986, Home Office Project License number PPL 70/7349, Dr. Isabelle Leclerc]. Procedures were performed at the Central Biomedical Service at Imperial College, London. Isolation of islets from multiorgan donors was approved by the local ethics committee at the University of Pisa. Human pancreata were collected from brain-dead organ donors after informed consent was obtained in writing from family members. Use of human islets at Imperial College was approved by the local NRES Committee, Fulham; REC reference 07/H0711/114.

#### Expression quantitative trait loci analysis.

Human islet DNA samples obtained from 53 donors (see Supplementary Table 1 online for clinical characteristics), using the DNeasy Blood & Tissue Kit (QIAGEN, Hilden, Germany) as previously described (51), were genotyped for SNPs rs4502156, rs7172432, and rs7163757. The rs7172432 locus was amplified by semi-nested PCR using primers TAG GTA TCT TGG AGC TGA GG and CCA CAC TTC ACA GAA TCA GG for the first round amplification and then CAG GTC AAG TGA GCA CTT GC and CCA CAC TTC ACA GAA TCA GG for the second round. The amplicons were then digested with *Ssp*I and genotyped based on the resulting restriction fragment length polymorphism.

Islet RNA was isolated from hand-picked islets as described (39), using the Arcturus PicoPure RNA Isolation Kit (Applied Biosystems, Foster City, CA), according to the procedure recommended by the manufacturer for RNA extraction from cell pellets and was accordingly treated with DNase to remove the contamination with genomic DNA. Reverse transcription to cDNA was performed using a High Capacity cDNA Reverse Transcription Kit (Themofisher). The rs4502156 and rs7163757 SNPs were genotyped by qPCR using a commercial TaqMan assay (Applied Biosystems). VPS13C, C2CD4A, and C2CD4B mRNA abundances were measured relative to ACTB in corresponding RNA samples by qRT-PCR using commercial TaqMan assays (Applied Biosystems) and the ΔC_T_ method. As a quality control step, samples with ΔC_T_ standard deviation > 0.2 were excluded from the analysis. The association between VPS13C expression and genotype was tested using an ANCOVA model, controlling for age, sex, and BMI and implemented in R (52). The association of genotype with C2CD4A and C2CD4B was analyzed in the same manner. Linkage disequilibrium (LD) values for SNPs in the Tuscan population used here were obtained at: http://www.1000genomes.org/faq/which-populations-are-part-your-study.

#### Luciferase construct cloning and assay.

To assess whether variants at rs7163757 might cause changes in the expression of nearby genes, two reporter constructs were generated. A 1.3-kb fragment of the genomic region flanking the SNP was amplified by PCR from a heterozygous donor by using Phusion High Fidelity DNA Polymerase (Thermo Scientific, Paisley, UK). The PCR product was subsequently cloned into CR™8/GW/TOPO (Thermofisher, Paisley, UK) according to the manufacturer's instructions. Plasmid DNA from clones was purified using a GenElute Plasmid Miniprep Kit (Sigma, Dorset, UK) and sent for sequencing to identify clones containing one of each allele. DNA fragments were then shuttled into the minimal promoter (DNA sequence: TAG AGG GTA TAT AAT GGA AGC TCG ACT TCC AG, containing a TATA box promoter element)-driven luciferase vector GL4.23-GW vector (76) using the Gateway LR Clonase II Enzyme Mix (Invitrogen, Paisley, UK). pGL4.23-GW is modified from pGL4.32 (Promega) with Gateway technology (Thermofisher) and has previously been used successfully for the analysis of enhancer activity (20).

The sequence and orientation of the insert was checked by restriction enzyme digest, and subsequently a QIAGEN Plasmid Maxi Kit (QIAGEN, Manchester, UK) was used to purify transfection grade DNA. HEK293, MIN6 (44), 1.1B4 (41), and EndoC-βH1 (54) cells were transfected using Lipofectamine 2000 (Invitrogen, Paisley, UK) in 48-well plates using 250 ng of each reporter construct and 1 ng of Renilla control vector. Each condition was repeated in six separate wells. Dual-Luciferase Reporter Assay (Promega, Southampton, UK) was used to measure Luciferase normalized against Renilla. All experiments were done in triplicate. The following cloning primers were used: CCA ACA AAT AGT AAG CAT TAT TAC C (rs7163757, forward) and CAA ATA GTT GTA GAT ATG TGG CAT T (rs7163757, reverse).

#### Mouse generation, housing, and genotyping.

Generation of heterozygous embryos on a C57/BL6 background, carrying floxed alleles of Vps13c was conducted by GenOway (France). Vps13c^fl/fl^ mice were crossed to mice expressing Cre recombinase under the control of the Ins1 promoter (33, 69) to generate mice in which exon 1 of the Vps13c gene was selectively excised in pancreatic β-cells. Mice were born at the expected Mendelian ratios without any obvious physical or behavioral defects. Mice were housed two to five per cage in a pathogen-free facility under a 12:12-h light-dark cycle and had ad libitum access to water and standard mouse chow diet (Research Diet, New Brunswick, NJ). High-fat diet (HFD, 60% wt/wt fat content; Research Diet) was introduced at 4 wk of age.

Genotyping was performed from ear biopsies using PCR. Knockout of Vps13c from pancreatic islets was assessed using both qPCR and immunoblotting, as described below. Mice were weighed weekly from 5 wk of age, and random, fed glycemia was tested fortnightly in the afternoon.

#### Intraperitoneal and oral glucose tolerance tests.

Mice were fasted for 15–16 h overnight prior to intraperitoneal (IPGTT) and oral glucose tolerance tests (OGTT) with free access to water. Blood samples were taken for glycemia measurement via venesection of the tail vein. Glycemia was measured using an Accu-Chek glucometer (Roche Diabetes Care, UK) and appropriate measurement strips. Fasting glycemia was first measured (*time 0*), and then glucose was administered via ip injection (1 g/kg body wt) or oral gavage (1.5 g/kg body wt). Glycemia measurements were then taken by injection at 15, 30, 45, 60, 90, and 120 min.

#### Measurement of plasma insulin and proinsulin.

Mice were fasted overnight with free access to water. A fasting (*time 0*) blood sample (∼50 μl) was collected from the tail vein into a lithium-heparin-lined Microvette (Starstedt, Leicester, UK) before administering of glucose (3 g/kg body wt) via ip injection. Blood samples were then collected at 15 and 30 min after injection. Glycemia was also measured at these time points. Plasma was collected by centrifuging samples at 2,000 *g* for 10 min at 4°C. Plasma insulin was measured using an ultrasensitive mouse insulin ELISA (Crystal Chem, IL). For random-fed insulin/proinsulin ratio measurements, a blood sample was collected into a lithium-heparin-lined Microvette from the tail vein and the aorta immediately after culling via cervical dislocation. Samples were kept on ice at all times to prevent degradation of proinsulin, and plasma was collected as described above. Insulin was measured as described above, and proinsulin was measured using a Rat/Mouse Proinsulin ELISA (Mercodia, Uppsala, Sweden).

#### Homeostatic model assessment analysis.

Homeostatic model assessment analysis (HOMA2)-%S and-%B (36) were calculated using fasting glycemia and plasma insulin measurements, with the HOMA Calculator, as described (https://www.dtu.ox.ac.uk/homacalculator/download.php).

#### Isolation of islets and assay of insulin secretion.

Mice were culled by cervical dislocation. Islets were isolated after pancreatic distension with collagenase essentially as previously described (55). Islets were allowed to recover from digestion for 24 h (RC-fed mice) or 48 h (HFD-fed mice) in RPMI medium (GIBCO) supplemented with 10% (vol/vol) fetal bovine serum, 1% (wt/vol) penicillin, 1% (wt/vol) streptomycin, 11.1 mM glucose, and 2 mM l-glutamine. Insulin secretion was measured from duplicate batches of 10 islets incubated in 0.5 ml of Krebs-Ringer medium [130 mM NaCl, 3.6 mM KCl, 1.5 mM CaCl_2_, 0.5 mM MgSO_4_, 0.5 mM NaH_2_PO_4_, 2 mM NaHCO_3_, 10 mM HEPES, and 0.1% (wt/vol) BSA, pH 7.4] containing 3 or 16.7 mM glucose or 20 mM KCl and 3 mM glucose as indicated, and shaking at 37°C for 30 min. Total insulin was extracted into 0.5 ml of acidified ethanol [75% (vol/vol) ethanol, 1.5% (vol/vol) 1 M HCl and 0.1% (vol/vol) Triton X-100]. For continuous measurements of secretion, insulin samples from 50 perifused islets were collected using a custom-built device and a perifusion rate of 500 μl/min at 37°C as described previously (10). Secreted and total insulin concentrations were measured using a homogeneous time-resolved fluorescence-based (HTRF) insulin assay (CisBio, Codolet, France) in a PHERAstar reader (BMG Labtech), according to the manufacturer's instructions.

#### Determination of β- and α-cell mass.

Isolated pancreata were fixed in 10% (vol/vol) formalin overnight at 4°C and embedded in paraffin wax. Sections (5 μm) were cut and fixed onto Superfrost slides. For staining, five sections per mouse, 25 μm apart, were incubated in Histochoice Clearing Agent and then submerged consecutively in 100, 95, and 70% ethanol to remove the wax. Following washes with water, sections were permeabilized by boiling in a citrate-based antigen unmasking solution (Vector Labs, Peterborough, UK), washed with PBS, and then incubated in PBS-Triton X-100 [PBST, 0.1% (vol/vol)] containing 2% (wt/vol) BSA and 2% (vol/vol) goat and donkey serum for 2 h at room temperature. Sections were then incubated in a humidified chamber at 4°C overnight with guinea pig anti-insulin (1:200; Dako, Ely, UK) and mouse anti-glucagon (1:1,000; Sigma, Dorset, UK). After washing three times in PBST [0.25% (vol/vol)] containing 0.25% (wt/vol) BSA, sections were incubated with Alexa fluor 488 and 568-conjugated secondary antibodies (1:1,000; Invitrogen, Paisley, UK) for 2 h at room temperature in the dark. Sections were then mounted using Vectashield antifade mounting medium containing DAPI (Vector Labs). Slices were imaged in the Imperial College facility for imaging by light microscopy (FILM) (http://www3.imperial.ac.uk/imagingfacility), using a Zeiss Axio Observer inverted widefield microscope with LED illumination. Images were captured with a Hamamatsu Flash 4.0 fast camera controlled by Zen software (Zeiss, Cambridge, UK). Image analysis was conducted using ImageJ software (1) and an in-house macro as described under supplementary methods (see online).

#### Quantitative real-time PCR analysis.

Total RNA was extracted from 50–200 islets isolated from three control and three βVps13cKO mice (for both males and females) using TRIzol (ThermoFisher Scientific) according to the manufacturer's instructions. RNA (100 ng) was reverse transcribed to produce cDNA by using the High Capacity Reverse Transcription Kit (Life Technologies, Paisley, UK) with random primers. qPCR was conducted using SYBER Green (life Technologies, Paisley, UK) on an ABI-Fast Prism 7500 machine and primers specific to murine Vps13c, C2cd4a, C2cd4b, or cyclophilin A. Primers were designed using Primer Express 3.0 (Applied Biosystems, CA), and sequences used were: Vps13c forward CAC AAG CAT TGA AGA TAG AAG CAA AA, reverse AGT GAT GGC ACA ATG TCT TGT TG; C2cd4a forward CGG GTT GGA AAA CCA TCT GA, reverse GTC TGA ACC CTG TGA TCC TGT TC; C2cd4b forward ACG TCA CCT GCT TCG TTC CT, reverse CAC GAG CGT CTT TTC TTC TTC A; cyclophilin A forward TAT CTG CAC TGC CAA GAC TG A, reverse CCA CAA TGC TCA TGC CTT CTT TCA. Whereas the VPS13C and C2CD4B primers spanned exon/exon junctions, the C2CD4A primers spanned intron 1.

#### Western immunoblotting.

Total protein was extracted from 50–500 islets isolated from two or three control or β-cell-specific VPS13C knockout (βVps13cKO) mice (males and females) in ice-cold RIPA buffer [1% (vol/vol) Triton X-100, 1% (wt/vol) sodium deoxycholate, 0.1% (wt/vol) SDS, 0.15 M NaCl, 50 mM Tris, pH 8.0] containing a 2× concentration of Complete, EDTA-free protease inhibitor cocktail (Roche, Burgess Hill, UK). The samples were incubated in RIPA on ice for 10 min and then freeze-thawed twice to ensure release of proteins. Samples were clarified by centrifuging at 16,000 *g* for 10 min at 4°C, and then total protein content was quantified using a BCA protein assay kit (Pierce, ThermoScientific). Total protein (5 μg) was added to SDS sample buffer [0.5 M Tris·HCl, pH 6.8, 2% (wt/vol) SDS, 5% (wt/vol) glycerol, 0.6 M DTT, and 0.2 mg bromophenol blue] and incubated at room temperature for 30 min. Samples were then electrophoresed on a 4–10% discontinuous gradient gel alongside a HiMark Protein Standard (Novex; ThermoScientific) and transferred onto a nitrocellulose membrane overnight. Membranes were blocked with 5% milk and incubated with primary antibodies (rabbit anti-VPS13C, 1:2,000, described above, or goat anti-EEA1, Santa Cruz, TX, 1:2,000) overnight with agitation at 4°C. Membranes were then washed three times in PBS-Tween 20 (0.2% vol/vol) and incubated with horseradish peroxidase-conjugated antibodies for 1 h at room temperature. Following three washes in PBS-Tween 20, proteins were visualized with ECL reagent and X-ray film (Amersham, GE Healthcare Life Sciences).

#### Confocal immunocytochemistry.

Human islets were dissociated by 10-min incubation in Hanks'-based enzyme-free cell dissociation buffer (GIBCO, Invitrogen) and gentle pipetting to generate small clusters of cells. Dissociated cells were plated onto 13/24-mm sterile coverslips and allowed to recover for 1–2 days. Cells were fixed in 4% paraformaldehyde and permeabilized in 0.1% Triton X-100. Primary cells were blocked in 10% fetal calf serum and subsequently incubated overnight with VPS13C 15-E antibody (Santa Cruz sc-104751, 1:50) with or without anti-insulin antibody (1:200; Dako, Ely, UK) followed by incubations with Alexa 488 and Alexa 568-conjugated secondary antibodies in sequential order. Coverslips were mounted using VectaShield with DAPI and imaged as described elsewhere (42). Samples were illuminated using steady-state 488- and 560-nm laser lines, and emission was collected through ET535/30 and ET620/60 emission filters (Chroma). Images were captured using a Hamamatsu EM CCD digital camera controlled by an Improvision/Nokigawa spinning disc system running Volocit (PerkinElmer, MA) software.

#### Confocal Ca^2+^ imaging.

Islets were isolated as described above. Simultaneous imaging of Ca^2+^ of individual cells was performed by spinning disc confocal microscopy after loading intact islets with Fluo 2-AM (Cambridge Bioscience, Cambridge, UK). Images were captured with a Zeiss Axiovert 200M microscope fitted with a ×10 0.3–0.5 NA, EC Plan Neofluar, Zeiss objective and a ×1.5 Optivar attached to a Nokigawa spinning disc confocal head, as described (27). The microscope was controlled using Volocity software. Islets were continuously perifused in Krebs-Ringer buffer containing 3 mM glucose, equilibrated with 95% O_2_-5% CO_2_ at 34–36°C. Islets were stimulated at 210 s and 1,300 s by perifusion with Krebs-Ringer supplemented with up to 16.7 mM glucose or 20 mM KCl as indicated. Offline processing and analysis were conducted using ImageJ software (1) and an in-house macro as described under supplementary methods.

#### Statistics.

Data were analyzed using Microsoft Excel, GraphPad PRISM 6.0, and R. Significance was tested using an unpaired Student's two-tailed *t*-test with appropriate posttests for multiple comparisons, or two-way ANOVA, as indicated. *P* < 0.05 was considered significant, and errors signify means ± SE unless otherwise stated. Figures were constructed using Adobe Illustrator.

## RESULTS

### 

#### eQTL analysis.

**Fig. 1.**
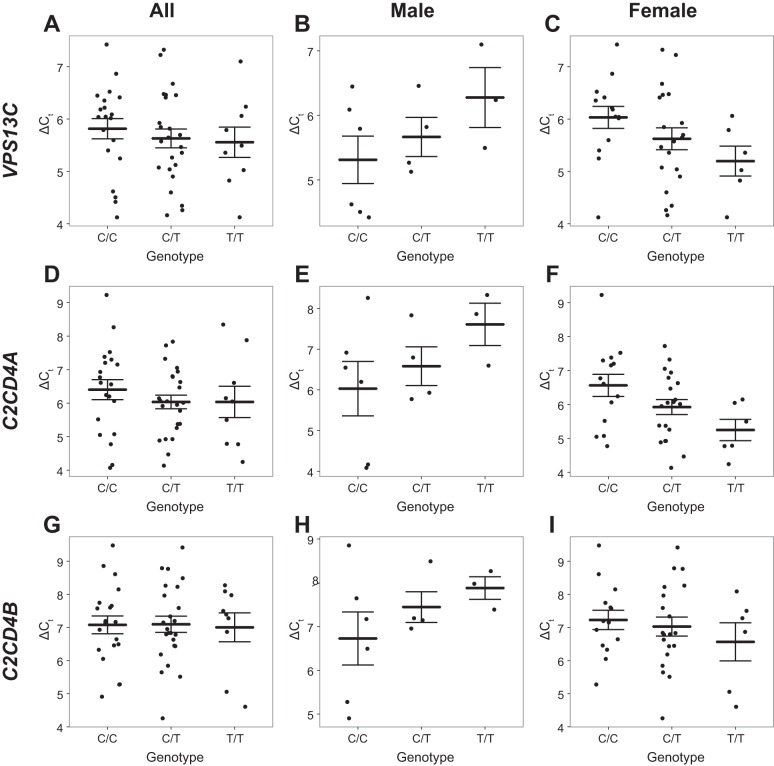
eQTL (expression quantitative trait loci) analysis. Expression of VPS13C (*A–C*), C2CD4A (*D–F*), and C2CD4B (*G–I*) was quantified relative to ACTB in 53 human donor islet samples and compared with the genotype at rs7163757. ΔC_T_ is plotted against genotype for all samples (*A, D, G*; *n* = 53) or just samples from male (*B, E, H*; *n* = 13) or female (*C, F, I*; *n* = 40) donors, along with the mean and standard error. Since higher ΔC_T_ corresponds to lower expression, possession of the risk allele (C) is significantly associated with lower VPS13C expression in samples from female donors (*P* = 0.041).

GWAS studies have implicated SNPs close to VPS13C, C2CD4A, and C2CD4B in altered T2D susceptibility. We tested the association between genotype at one of the previously identified SNPs rs4502156 and the likely causal SNP rs7163757 (61, 66) (*r*^2^ = 0.939, D′ = 0.979 with rs4502156) and VPS13C expression in human islet samples from 53 donors. Initial analysis for rs4502156 and rs7163757 including all samples showed no significant association. Interaction plots indicated a possible interaction between sex and genotype, which was tested by including the interaction term in the ANCOVA model (see materials and methods). This was found to be significant (*P* = 0.015, *n* = 53), so data were stratified by sex, and subsequently males and females were analyzed separately (Fig. 1, *A–C*). Analysis of females revealed a significant association between possession of the risk allele (C) at rs7163757 and lowered VPS13C expression (*P* = 0.041, *n* = 40; Fig. 1*C*). A similar sex interaction (*P* = 0.016, *n* = 53) was also observed for rs4502156, and likewise a significant association was detected between genotype at this locus and expression of VPS13C in females (*P* = 0.043, *n* = 40). An association was also detected between rs4502156 (not shown), as well as rs7163757 (*P* = 0.011, *n* = 40; Fig. 1, *D–F*), with C2CD4A mRNA levels in female donors but not with C2CD4B (Fig. 1, *G–I*). Subsequent functional studies in the present report focussed upon VPS13C.

#### Impact of risk alleles on enhancer activity assessed by reporter luciferase assay.

**Fig. 2.**
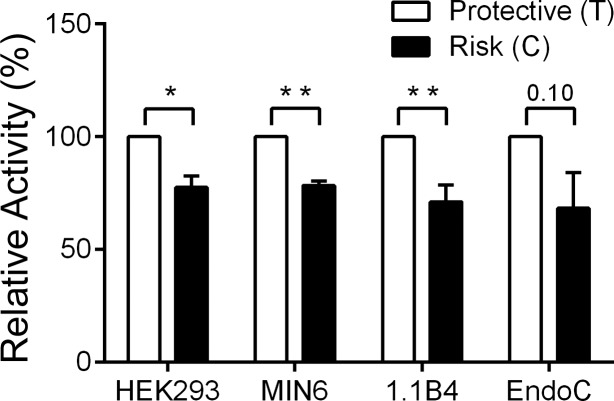
Comparison of promoter/enhancer activities of variants at rs7163757 in the VPS13C locus. Luciferase reporter assay performed in 4 cell lines (HEK293, MIN6, 1.1B4, and EndoC-βH1). The risk single nucleotide polymorphisms (SNP) caused a significant reduction in enhancer activity in HEK293, MIN6, and 1.1B4 (*P < 0.05, **P < 0.01 calculated using ratio paired Student's *t*-tests). Error bars represent SE from either 3 (HEK293 and MIN6) or 4 (1.1B4 and EndoC-βH1) independent experiments. *P* = 0.1 for the effect of the risk allele in EndoC-βH1 cells.

To determine whether and how the possession of risk alleles at the VPS13C locus might affect the expression of nearby or remotely located genes, we used reporter-luciferase assays in non-β-cells (HEK293) and in β-cell lines from mice (MIN6) and humans (1.1B4 and EndoCβH1). As shown in Fig. 2, inclusion of the risk (C) allele at the previously implicated causal SNP rs7163757 significantly lowered the enhancer/promoter activity of reporter constructs bearing this variant vs. the presence of the protective (T) allele in HEK293, MIN6, and 1.1B4 cells. A similar tendency (*P* < 0.1) was observed in EndoCβH1 cells (Fig. 2). These data are thus consistent with an enhancer function for this region, whose activity is lowered in carriers of risk alleles.

#### Impact of β-cell-selective deletion of Vps13c on body mass and fasting glycemia.

**Fig. 3.**
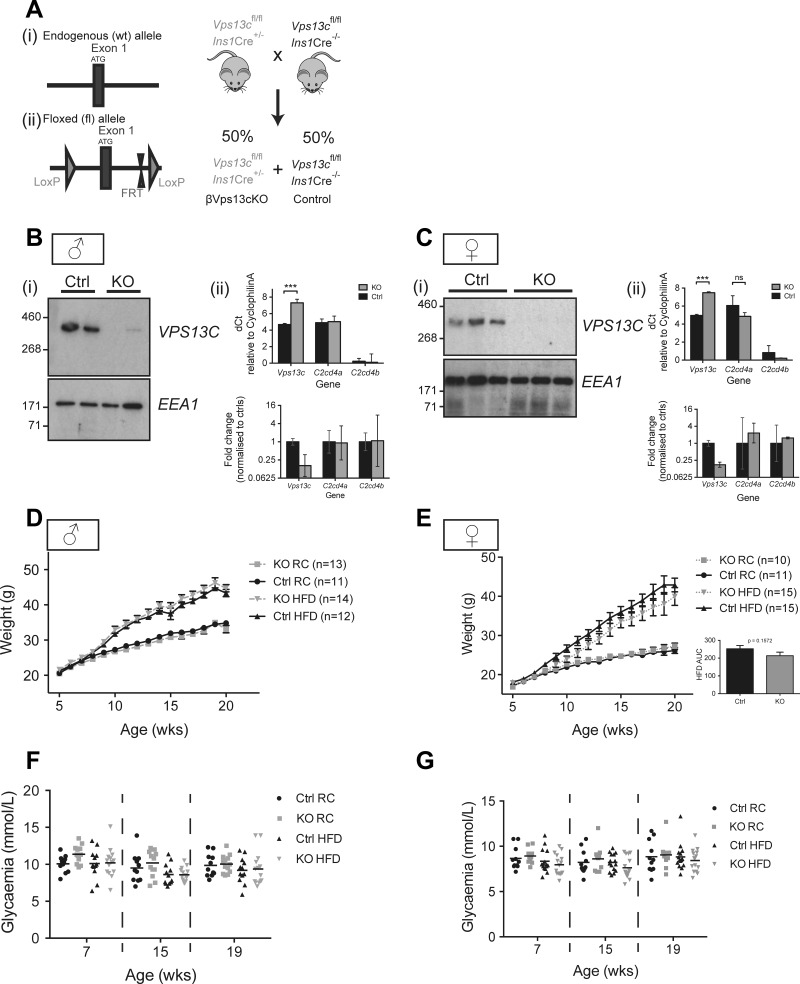
Generation of VPS13C^fl/fl^::Ins1.Cre^+/−^ (βVps13cKO) mice. *A*: LoxP sites were inserted on either side of exon 1 to enable Cre-mediated inactivation of the Vps13c gene in pancreatic β-cells after breeding to Ins1.Cre mice. The resultant colony consisted of VPS13C-null mice (KO, βVps13cKO) and control mice (Ctrl) at the expected 50:50 ratio. *B* and *C*: islets were isolated from 2–3 male (*B*) and 3 female (*C*) Ctrl and KO mice for (*i*) immunoblotting or (*ii*) qPCR analysis. Both ΔC_T_ (relative to cyclophilin A) and log2-transformed fold changes, normalized to control mice, are shown. Error bars represent standard deviation in (*ii*) *top* and 95% confidence intervals in (*ii*) *bottom*. *P < 0.05, **P < 0.01 analyzed with 2-way ANOVA with Sidak's multiple corrections. *D–G*: changes in weight (*D* and *E*) and random-fed glycemia (*F* and *G*) over time for Ctrl (black) and KO (dashed) mice fed regular chow (RC, circles or squares) or high-fat diet (HFD, triangles). *Inset*: area under the curve (AUC) analysis for female mice on HFD, assessed for significance using an unpaired Student's *t*-test; *n* = 11–15 mice, as indicated.

The observations above suggested that risk variants at the VPS13C locus may decrease the expression of nearby genes. To explore the potential impact of lowered VPS13C levels on insulin secretion, deletion of exon 1 (Fig. 3*A*) of the Vps13c gene was achieved throughout the pancreatic β-cell compartment in C57BL/6 mice from ∼E11.5 using the highly selective Ins1Cre deleter strain (69). As shown in Fig. 3, *B* and *C*, VPS13C was barely detected in islets isolated from βVps13cKO mice (Fig. 3*B* and *Ci*) and levels of Vps13c mRNA were significantly reduced (Fig. 3, *Bii* and *Cii*) by >80%. These findings are fully consistent with efficient (>94%) and exclusive (69) recombination in β-cells, which comprise 60–80% of the rodent islet (16), given that Vps13c mRNA is about twofold more abundant in β- than in α-cells (5), which comprise the majority of the islet non-β-cells. Expression of C2cd4a and C2cd4b in islets was variable between mice but was unaffected by Vps13c deletion. Changes in body weight gain (Fig. 3, *D* and *E*) and random-fed glycemia (Fig. 3, *F* and *G*) over time were not different between control (Ctrl) and βVps13cKO (KO) mice irrespective of sex or diet [regular chow (RC) vs. high-fat diet (HFD); see materials and methods for details].

#### βVps13cKO mice display age-dependent abnormalities in glucose tolerance.

**Fig. 4.**
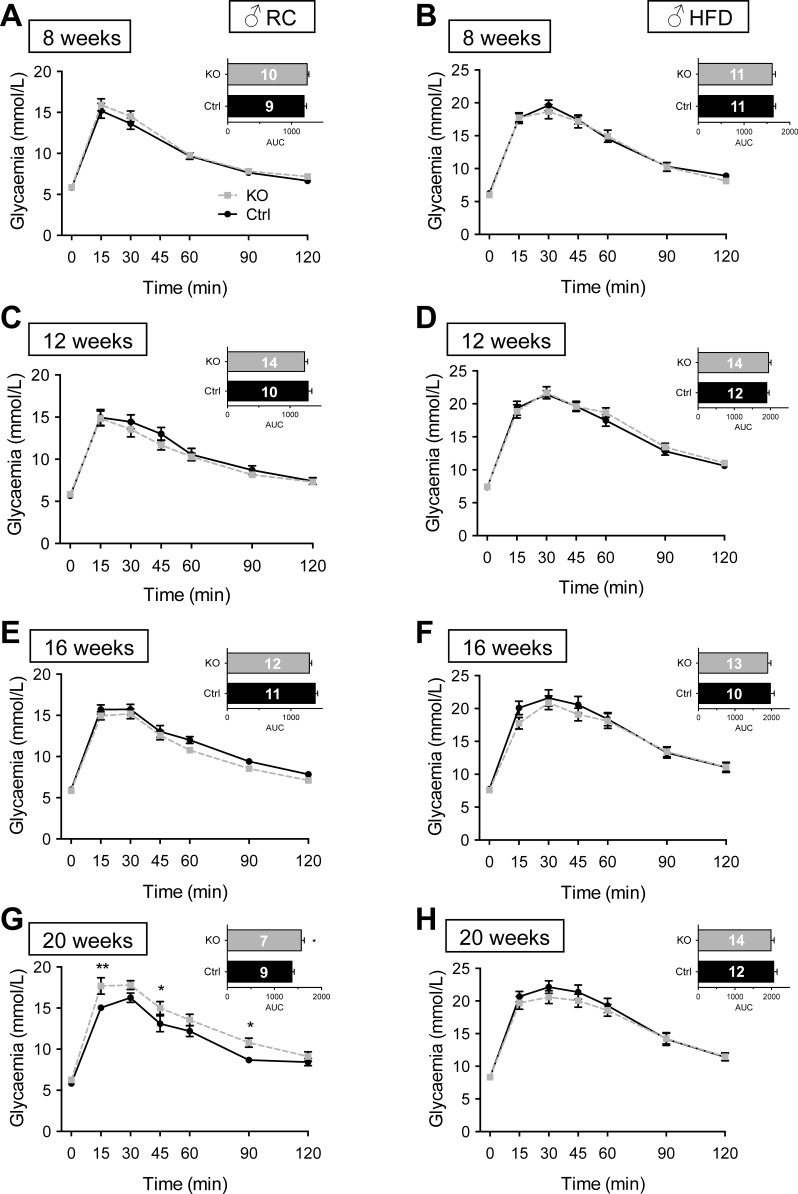
Glucose tolerance in male βVps13cKO mice. *A–H*: intraperitoneal glucose tolerance (1 g/kg body wt) was measured in Ctrl (solid black line) and KO (dashed line) male littermates fed either RC (*A, C, E, G*) or HFD (*B, D, F, H*). IPGTTs were conducted at 8 (*A, B*), 12 (*C, D*), 16 (*E, F*), and 20 (*G, H*) wk. *Inset*: AUC. Numbers of animals (*n*) for each experiment are given in AUC bars. *P < 0.05, **P < 0.01, 2-way ANOVA with Fisher's LSD post hoc test (main graphs) or unpaired Student's *t*-test (AUC, *insets*).

Examined in male mice, intraperitoneal glucose tolerance (IPGTT) was not different between control and βVps13cKO animals up to the age of 16 wk, whereas βVps13cKO mice became glucose intolerant at 20 wk of age (Fig. 4, *A, C, E, G*). Although glucose tolerance was lower at all ages examined compared with animals maintained on RC, no differences were observed between control and βVps13cKO males maintained for up to 16 wk (i.e., 20 wk old) on a HFD (Fig. 4, *B, D, F, H*).

**Fig. 5.**
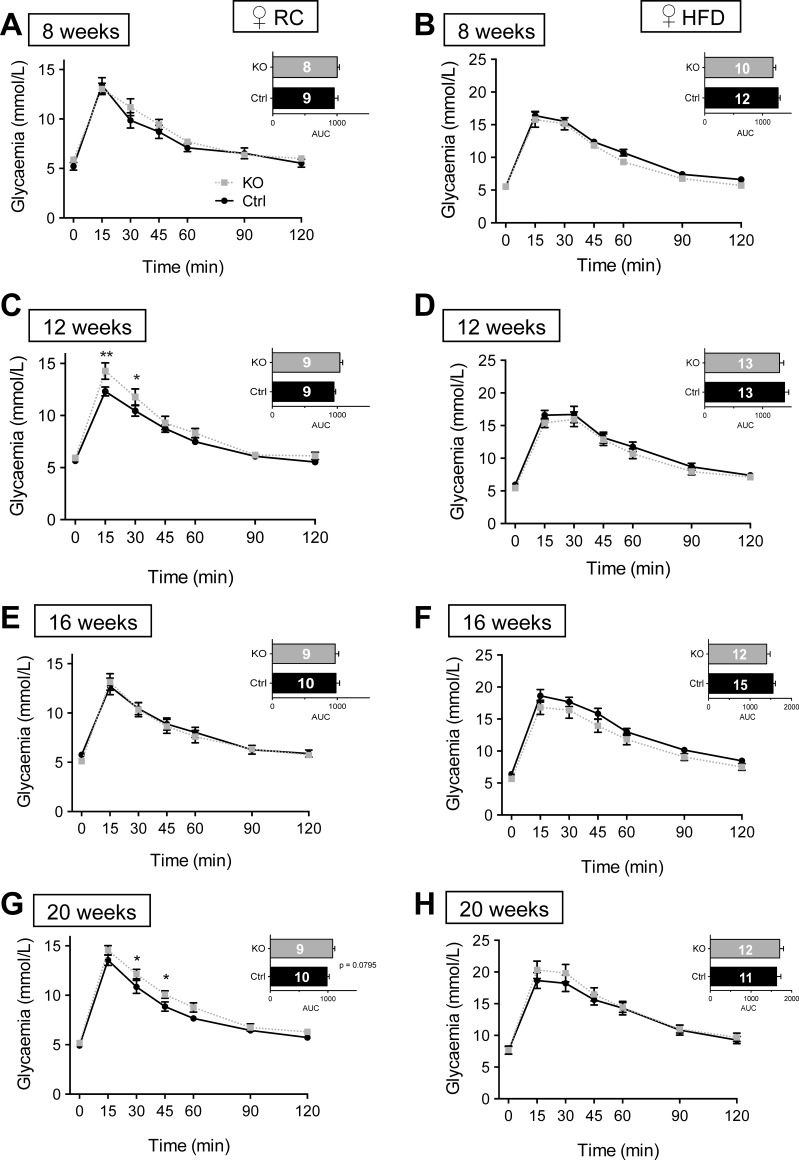
Glucose tolerance in female βVps13cKO mice. *A–H*: intraperitoneal glucose tolerance (1 g/kg body wt) was measured in Ctrl (solid black line) and KO (dotted line) female littermates fed RC (*A, C, E, G*) or HFD (*B, D, F, H*). IPGTTs were conducted at 8 (*A, B*), 12 (*C, D*), 16 (*E, F*), and 20 (*G, H*) wk. *Inset*: AUC. Numbers of animals (*n*) for each experiment are given in the AUC bars. *P < 0.05, 2-way ANOVA with Fisher's LSD post hoc test (main graphs) or unpaired Student's *t*-test (AUC, *insets*).

By contrast, when maintained on RC, female mice (Fig. 5) displayed abnormal IPGTT at 12 wk of age (Fig. 5*C*). This resolved at 16 wk but was again apparent at 20 wk (Fig. 5*G*). Consistent with observations in males (Fig. 4), female βVps13cKO mice fed HFD similarly failed to show abnormalities in IPGTT up to 20 wk of age (Fig. 5, *B, D, F, H*).

**Fig. 6.**
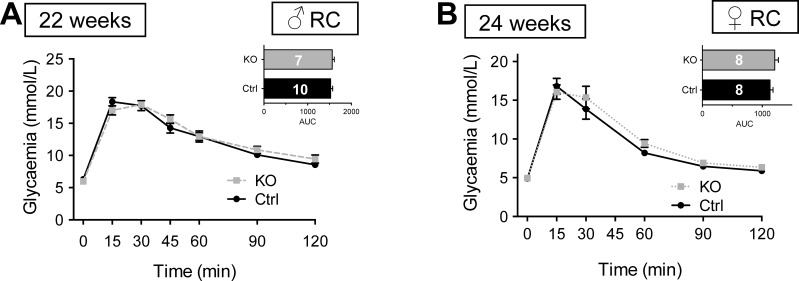
Oral glucose tolerance in βVps13cKO and control mice. *A* and *B*: oral glucose tolerance (1.5 g/kg body wt) was measured in Ctrl (solid black line) and KO [dashed (male) or dotted (female) lines] littermates fed RC. OGTTs were conducted at ages indicated. *Inset*: AUC; *n*, numbers for each experiment are given in AUC bars. *P < 0.05, 2-way ANOVA with Fisher's LSD post hoc test.

To test a possible role for Vps13c in responses to incretin hormones, we next performed oral glucose tolerance tests (OGTT) in RC-fed mice. No genotype-dependent differences were apparent in males (Fig. 6*A*) or females (Fig. 6*B*) examined at 22–24 wk.

**Fig. 7.**
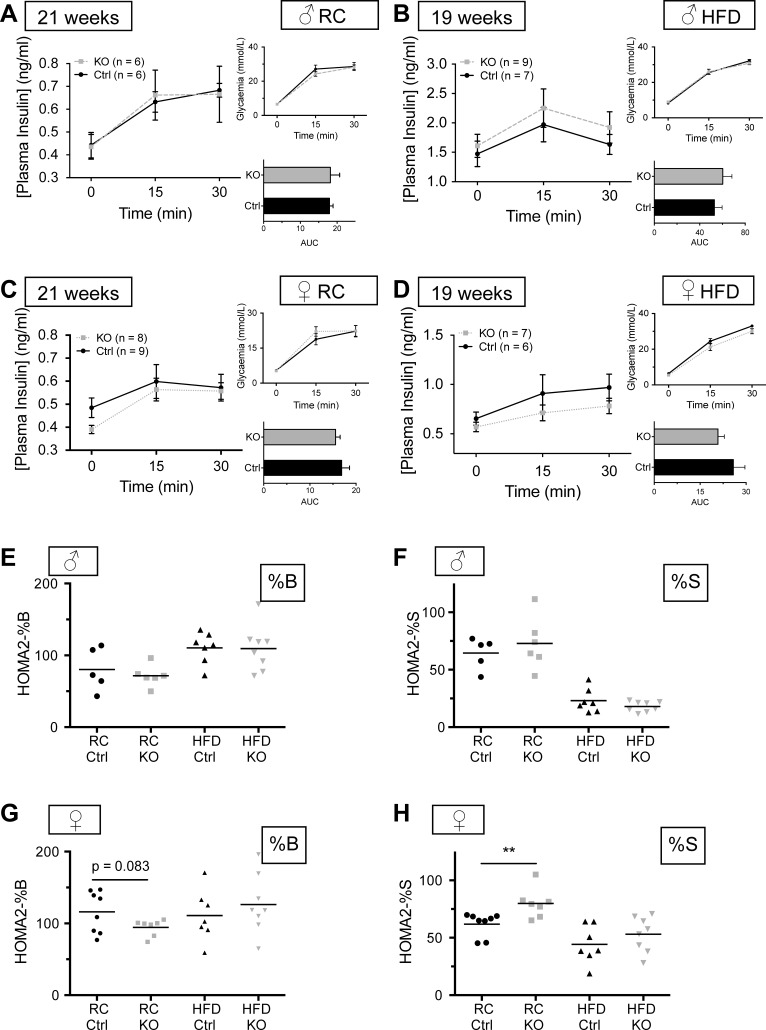
Effect of Vps13C deletion on glucose-stimulated insulin secretion (GSIS) in vivo. *A–D*: plasma insulin concentration was measured following intraperitoneal administration of glucose (3 g/kg body wt) in Ctrl (solid black line) and KO [dashed (males) or dotted lines (females)] littermates. Blood was sampled for insulin measurements when mice were 21 or 19 wk old (RC or HFD, respectively). *Inset, top*: respective glycemia measurements. *Inset, bottom*: AUC calculated from the main graph, measuring total released plasma insulin; *n* = 6–9 mice per genotype, as detailed in the key. *E–H*: homeostatic model assessment analysis (HOMA2)-%B (*E* and *G*) and -%S (*F* and *H*) analysis using fasting glycemia values and corresponding plasma insulin concentrations, respectively. **P < 0.01, unpaired Student's *t*-test with Welch's correction (*E–H*).

Examined in mice aged 19–21 wk, glucose-induced excursions in plasma insulin were not different between βVps13cKO and control male and female mice (Fig. 7, *A–D*). Likewise, by analyzing fasting glucose and insulin levels, we observed no indication of a change in steady-state β-cell function (36) as assessed using homeostatic model assessment (HOMA2-%B; Fig. 7*E*), nor insulin in insulin sensitivity (HOMA2-%S; Fig. 7*F*) in males maintained on either RC or HFD. By contrast, a tendency toward a lower HOMA2-%B value (Fig. 7*G*), accompanied by a significant increase in HOMA2-%S, was apparent in female βVps13cKO mice vs. controls fed on RC, whereas these differences were not observed on HFD (Fig. 7, *G* and *H*).

#### Impact of Vps13c deletion on glucose- and KCl-stimulated insulin secretion in vitro.

**Fig. 8.**
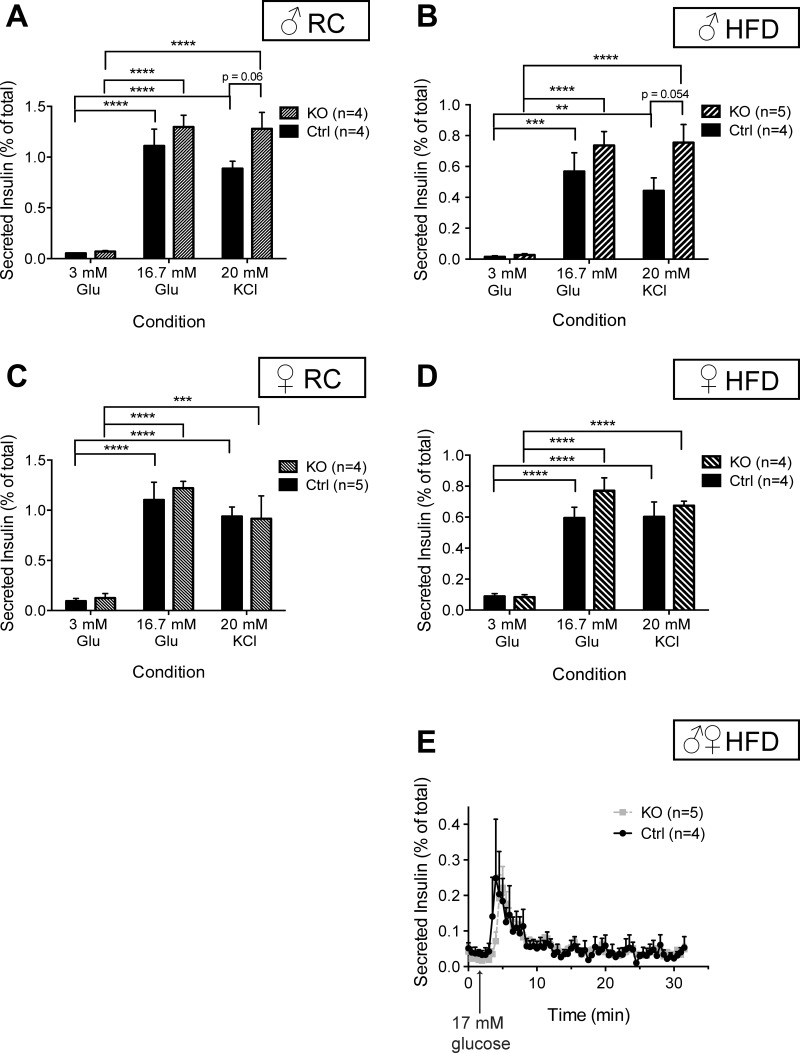
Effect of Vps13c deletion on GSIS in vitro. *A–D*: insulin secretion from isolated islets from KO and Ctrl mice over 20 wk old maintained on RC (*A* and *C*) or HFD (*B* and *D*) was assessed by incubating 10 size-matched islets in Krebs-Ringer solution containing 3 mM glucose (3 Glu), 16.7 mM glucose (16.7 Glu), or 20 mM KCl for 30 min and measuring the amount of insulin secreted (see materials and methods). Islets were lysed to measure total insulin; results are presented as %total insulin. *E*: insulin secretion from islets continuously perifused with Krebs-Ringer solution containing 3 mM glucose and then stimulated with 16.7 mM glucose; *n* = 3–5 mice per genotype, as indicated. *P < 0.05, **P < 0.01, ***P < 0.001, ****P < 0.0001, 2-way ANOVA with Sidak or Tukey's post hoc test where appropriate.

Impairments in glucose tolerance and a tendency toward impaired β-cell function apparent in vivo in female βVps13cKO mice might reflect abnormal glucose- or depolarization-dependent insulin secretion from β-cells. To investigate this, we studied insulin release from batches of islets from mice 20–23 wk old, as shown in Fig. 8. Interestingly, both glucose (16.7 mM) and KCl (20 mM) -stimulated secretion tended to increase in βVps13cKO vs. control islets from males fed either RC (Fig. 8*A*) or HFD (Fig. 8*B*). Whereas a similar tendency was also apparent for islets from females maintained on a HFD (Fig. 8*D*), those from female βVps13cKO mice fed RC showed no change in insulin secretion vs. controls (Fig. 8*C*) when stimulated with 20 mM KCl. No differences between HFD-fed control and βVps13cKO mice were seen when the same experiment was conducted under perifusion (Fig. 8*E*).

#### β-Cell mass is not changed after Vps13c deletion.

**Fig. 9.**
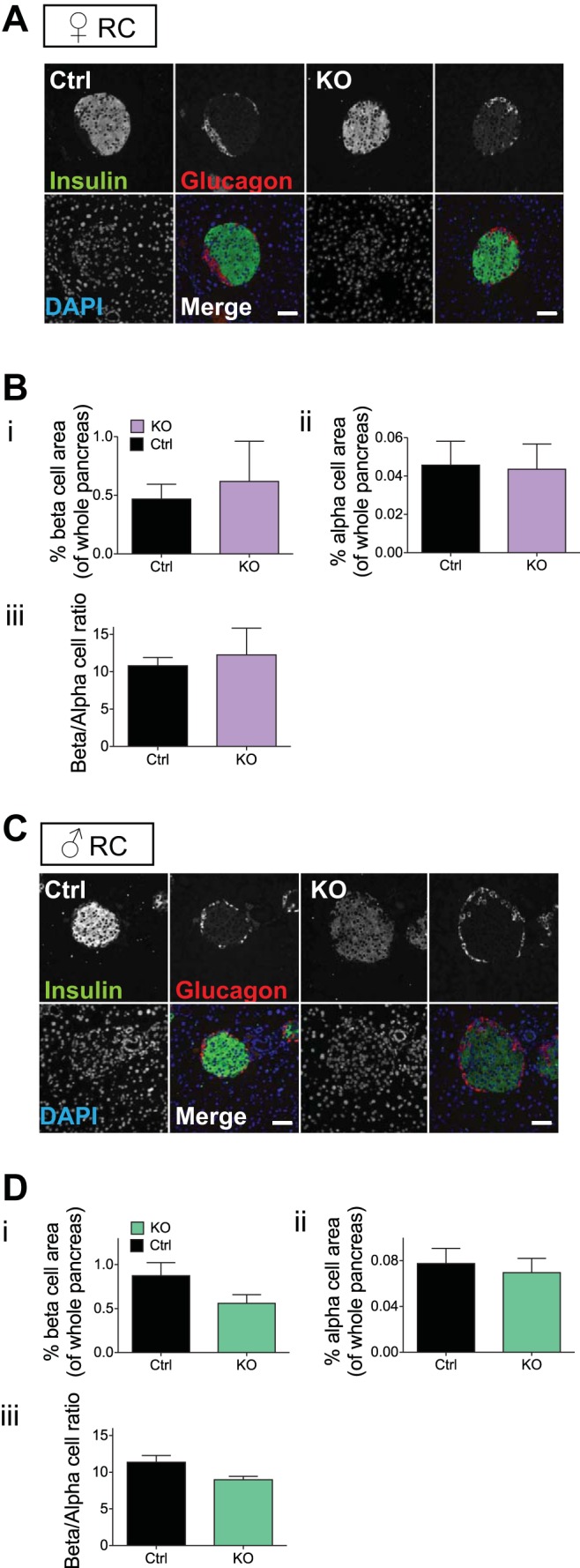
β-Cell mass in βVps13cKO mice. *A* and *C*: representative images from pancreatic slices from female (*A*) and male (*B*) Ctrl and KO mice (20–23 wk of age) fed a RC diet. Slices were stained with antibodies against insulin (green) and glucagon (red). Nuclei were stained with DAPI; scale bar represents 50 μm. *B* and *D*: percentage of β- (*i*) and α-cell (*ii*) surface area, normalized to whole pancreas surface area; (*iii*): β/α-cell ratio. Data are from *n* = 3 Ctrl and 3 KO females and 3 Ctrl and 5 KO males. No significant differences between genotypes were detected.

One explanation for the differences in glucose tolerance and insulin secretion seen in βVps13cKO mice may be an alteration in β-cell mass. To establish whether this was the case, we conducted immunohistochemical analyses on pancreatic sections from βVps13cKO and control mice fed RC and aged over 20 wk. Using antibodies against either insulin or glucagon, we observed no differences in %β- or α-cell surface normalized pancreatic surface (Fig. 9, *A* and *B*, females; *C* and *D*, males).

#### Sex-specific effects of Vps13c deletion on intracellular Ca^2+^ dynamics.

**Fig. 10.**
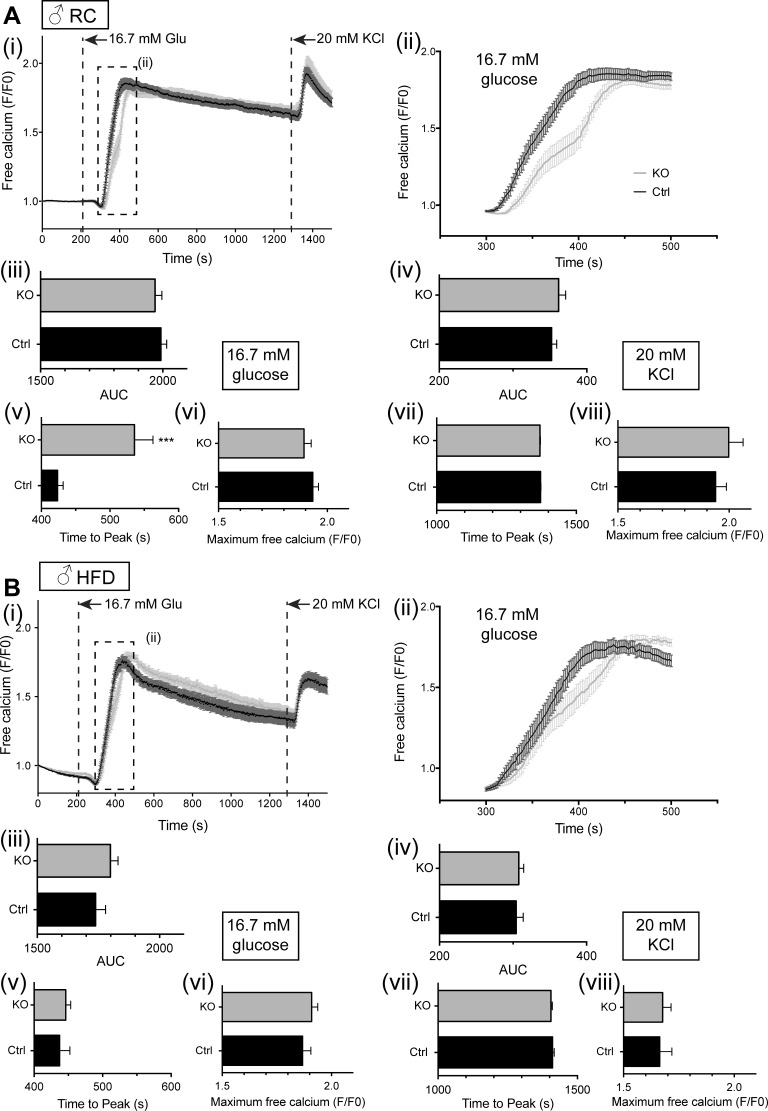
Effect of Vps13c deletion on calcium signaling in vitro in male mouse islets. Isolated islets from male mice (20–23 wk of age), maintained on RC (*A*) or HFD (*B*) were loaded with Fluo 2 and incubated in Krebs-Ringer solution containing 3 mM glucose (3 mM Glu) for 45 min. Dye-loaded islets (3–7 per field of view) were imaged on a spinning disk confocal microscope for 2 min in 3 mM glucose, as described in materials and methods. A perifusion system was used to allow subsequent imaging of the islets in 16.7 mM Glu for 18 min, followed by 20 mM KCl for 5 min. Individual traces from each islet were then averaged to give one trace per islet, which was then pooled with the other islets. (*i*), mean free Ca^2+^ (normalized to initial fluorescence; F/F0); (*ii*), *inset* from (*i*), mean free Ca^2+^ measured between 300 and 500 s, showing the effect of stimulation with 16.7 mM glucose; (*iii*), AUC analysis for high glucose stimulation; (*iv*), AUC analysis for KCl stimulation; (*v*), time to maximum peak value from stimulation with glucose; (*vi*), maximum peak value (F/F0) from stimulation with glucose; (*vii*), time to maximum peak value from stimulation with KCl; (*viii*), maximum peak value (F/F0) from stimulation with KCl; *n* = 3–5 mice per genotype. Number of islets used: *n* = male RC 31–38 islets from 3 mice; male HFD mice *n* = 41–46 islets from 4 mice. *P < 0.5, **P < 0.01, ***P < 0.001, unpaired Student's *t*-test.

**Fig. 11.**
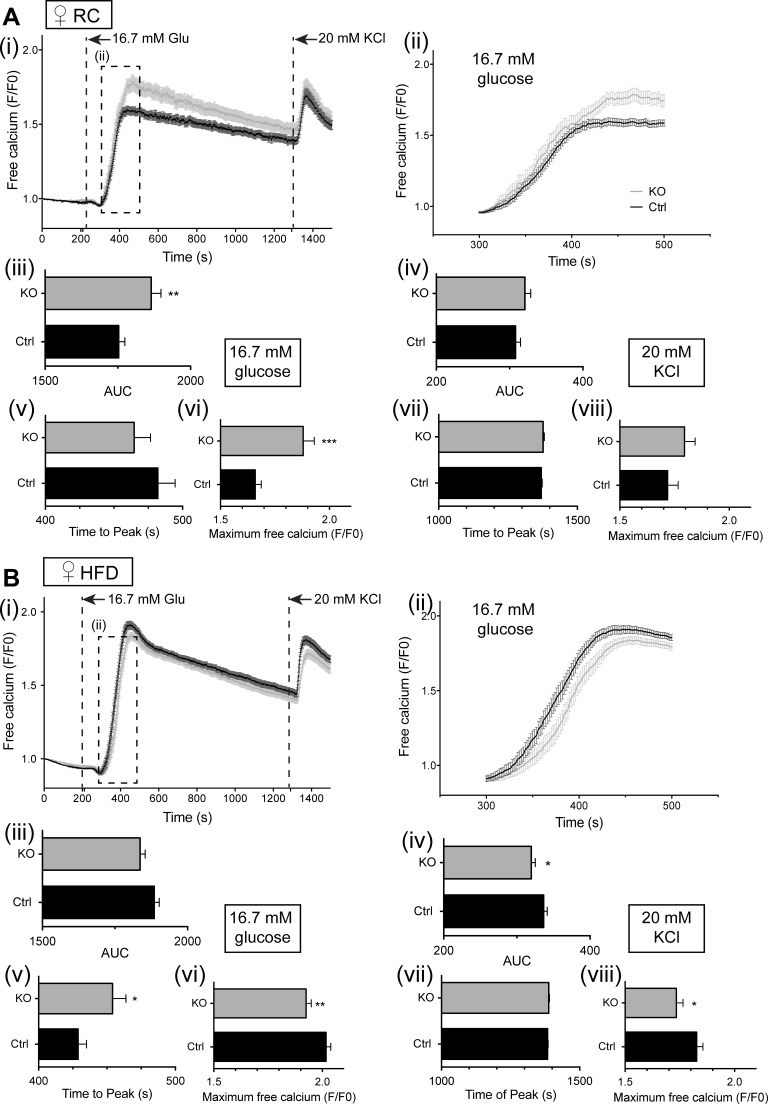
Effect of Vps13c deletion on calcium signaling in vitro in female mouse islets. Isolated islets from female mice (20–23 wk of age) maintained on RC (*A*) or HFD (*B*) were loaded with Fluo 2 and analyzed as for male islets (Fig. 10). (*i*), mean free calcium (F/F0); (*ii*), *inset* from (*i*), mean free calcium measured between 300 and 500 s, showing stimulation with 16.7 mM glucose; (*iii*), AUC analysis for high glucose stimulation; (*iv*), AUC analysis for KCl stimulation; (*v*), time to maximum peak value from stimulation with glucose; (*vi*), maximum peak value (F/F0) from stimulation with glucose; (*vii*), time to maximum peak value from stimulation with KCl; (*viii*), maximum peak value (F/F0) from stimulation with KCl; *n* = 3–5 mice per genotype. Number of islets used: female RC, *n* = 47–48 islets from 4 mice; female HFD, *n* = 46–59 islets from 4 or 5 mice (KO vs. Ctrl, respectively). *P < 0.5; **P < 0.01; ***P < 0.001; unpaired Student's *t*-test.

Alterations in glucose tolerance and tendency toward impaired insulin secretion, which were apparent in vivo, may reflect altered signal generation by glucose. We next used the fluorescent intracellular probe Fluo 2 (27) to monitor intracellular free Ca^2+^ dynamics in β-cells in situ within the intact islet (Figs. 10 and 11). Under the conditions used, glucose-induced changes in free Ca^2+^ were largely restricted to the β-cell population (10, 27). No genotype-dependent differences in the peaks of the Ca^2+^ response to either high glucose or KCl were apparent in islets from male mice (Fig. 10), although islets from male βVps13cKO mice fed RC did display a significantly delayed response to high glucose stimulation (Fig. 10*A, ii* and *v*). Increases in free Ca^2+^ in islets from female mice were respectively augmented (Fig. 11*A, i* and *vi*) and reduced (Fig. 11*B, i* and *vi*) in high-glucose-stimulated islets from βVps13cKO animals fed RC or HFD. A similar trend was seen after depolarization with KCl (Figs. 11*A, i* and *viii*, and *B*, *i, iv*, and *viii*). As was the case for male mice, the response to glucose in islets from HFD-fed female mice was slightly delayed, with those from RC mice showing no significant difference in the time of the glucose peak (Fig. 11*A, i* and *v*, and *B, i* and *v*).

#### Subcellular localization of VPS13C in human β-cells.

**Fig. 12.**
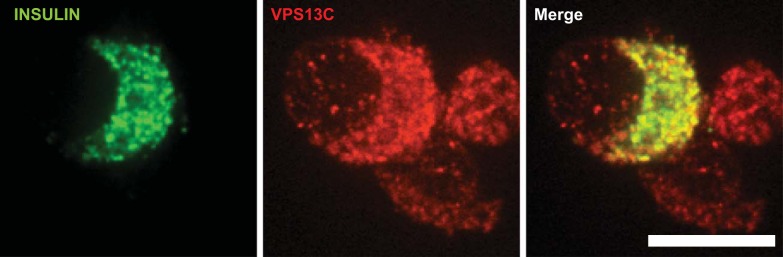
Subcellular localization of VPS13C in human β-cells. Immunocytochemical analysis for insulin (green) and VPS13C (red) was performed using confocal microscopy in single β-cells as described in materials and methods. Scale bar, 10 μm.

To determine whether VPS13C might conceivably affect the properties (i.e., “fusogenecity”), or the distribution of secretory granules, we explored the localization of the protein with single human β-cells by confocal immunocytochemistry (Fig. 12). Close colocalization was observed between insulin and VPS13C-labeled structures, indicative of the presence of the latter on the limiting membrane of insulin-containing dense core granules.

## DISCUSSION

Previous studies (17) have revealed that VPS13C expression in human islets is associated with HbA1c levels by massive parallel sequencing (RNA-seq) and microarray analysis at both nominal and permutation *P* values (*P* < 0.05), with lower mRNA levels observed in T2D subjects. We extend these findings here by showing that VPS13C mRNA levels were lower in carriers of risk alleles at rs4502156 and rs7163757 in female, but not male subjects. We note that in the present study a lower number of male vs. female samples may have limited our power to detect changes in the former. However, and arguing against this possibility, no tendency toward lowered VPS13C or C2CD4A expression with the interrogated SNPs was observed in males: rather, the trend was toward increased expression with risk alleles.

Using mouse genetics we provide evidence that VPS13C plays a role in the control of pancreatic β-cell function. It should be emphasized that the impact of deleting this gene selectively in the β-cell was relatively mild and indeed was not apparent in males until 20 wk of age. Evidence for deficiencies in β-cell function were, however, more apparent in females from an earlier age, in line with the human eQTL data. These included the transient appearance of glucose intolerance at 12 wk and its reemergence at 20 wk. Interestingly, the same phenomenon is also observed in a monogenic form of diabetes resulting from misexpression of the ZAC gene, termed transient neonatal diabetes mellitus (TNDM), and is apparent in mouse models with this disease (albeit in younger animals than observed here) (38). While the reasons for this transience are not known either in TNDM or in the case of Vps13c deletion, dynamic changes in the balance between islet function and insulin sensitivity may provide one explanation. Similarly, the emergence of glucose intolerance with age in βVps13cKO mice, which is reminiscent of changes seen after the inactivation of the T2D GWAS gene Tcf7l2 in mice (43, 82), seems to reflect, at least in part, increasing insulin resistance as well as impaired insulin output from the pancreas. Of note, recent studies report relatively preserved glucose sensing of isolated islets with age in both mice and humans (3, 26) but suggest a role for altered vascularization and fibrosis in impaired insulin secretion in vivo (3).

Strikingly, the impairments in glucose tolerance apparent in both male and female βVps13cKO mice vs. littermate controls at this age were abolished after maintenance on HFD. These findings demonstrate an interesting interaction between the inheritance of a genetic factor influencing risk, and age [as observed in human T2D, (87)] as well as sex and diet. The reasons for the difference in penetrance between the effects of Vps13c deletion observed here between male and female mice remain unknown but may reflect interactions with sex hormones at the level of the individual β-cell (46) or, alternatively, subtle differences in insulin sensitivity between the sexes that go on to influence the effect of perturbations in the β-cell on overall glucose homeostasis.

Examined in either males or females, β-cell mass was not different between control and knockout mice, indicating a possible defect in β-cell function as underlying the glucose dyshomeostasis reported above. Correspondingly, clear tendencies were apparent toward impaired β-cell function and lowered insulin levels when one combined fasting glycemia with corresponding insulin plasma concentration using HOMA2 analysis (particularly in females; Fig. 7, *E* and *G*). According to this analysis, insulin sensitivity was slightly but significantly increased in knockout females vs. littermate controls (Fig. 7*H*), again indicating that a defect in β-cell function is likely to underlie the mild glucose intolerance in knockout mice (and subject to caveats in extrapolating HOMA2 models from humans to rodents) (74).

On the other hand, we were unable to detect any impairment in glucose or depolarization-induced insulin secretion as assessed ex vivo in isolated islets (Fig. 8). Indeed, in islets isolated from animals maintained on either RC or HFD, we observed a tendency in male βVps13cKO mice toward enhanced insulin secretion in response to either high glucose or KCl and in female βVps13cKO in response to high glucose. By contrast, stimulated insulin secretion in response to KCl tended not to change in βVps13cKO vs. control islets from females fed RC or HFD. We are therefore unable at the present time to assign the changes in β-cell function and glucose homeostasis observed in vivo unambiguously to alterations in islet responses measureable in vitro. We would stress, however, that the mechanisms responsible for the stimulation of insulin secretion by elevated glucose in vivo, which are likely to be modulated by a multitude of humoral (e.g., circulating fatty acids, incretins, adipokines, etc.), neuronal (56), and other inputs into the islet, are unlikely to match perfectly those tested in vitro.

**Fig. 13.**
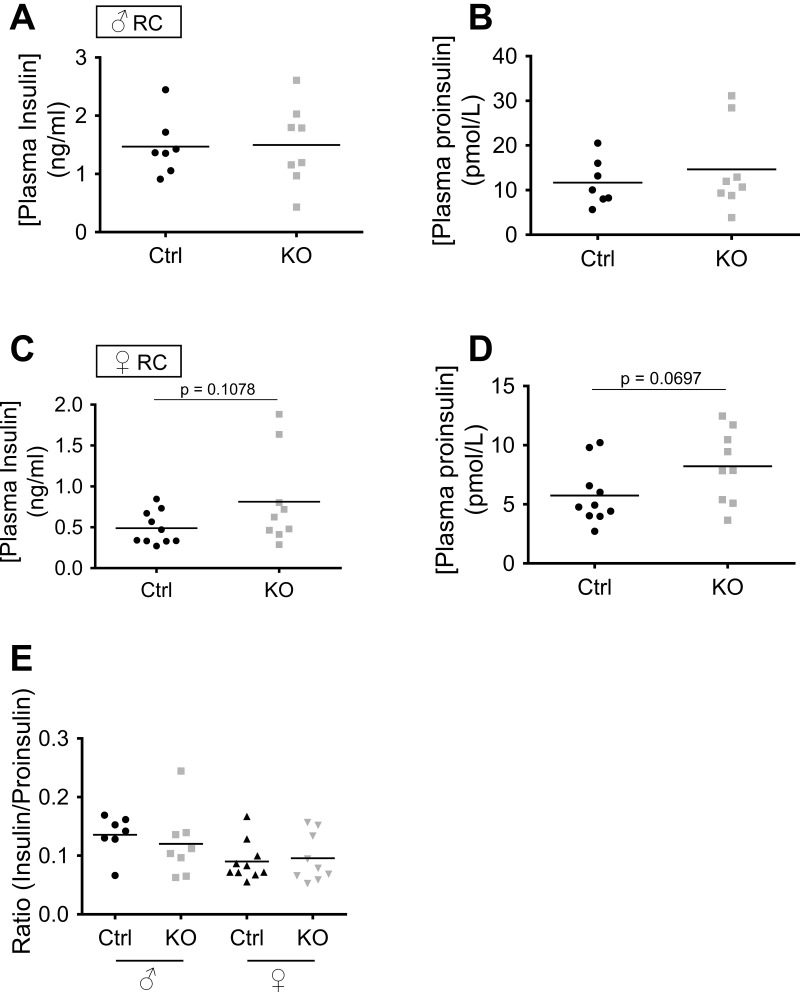
Random-fed insulin and proinsulin plasma concentrations in RC-fed mice. Plasma insulin and proinsulin were measured following collection from the tail vein (before culling) and aorta (immediately post mortem) of RC-fed Ctrl (black) or KO [green, males (*A* and *B*); purple, females (*C* and *D*)] mice aged over 21 wk. The ratio plasma insulin/plasma proinsulin is shown in *E*; *n* = 7 Ctrl and 8 KO (males), 10 Ctrl and 9 KO (females).

Nonetheless, detailed analysis of glucose- and KCl-induced Ca^2+^ dynamics did provide evidence for alterations at the level of secretory granule behavior, which may play a role to impair insulin secretion in vivo. Importantly, islets from male βVps13cKO mice maintained on RC or on HFD responded normally with insulin secretion in response to either glucose or high KCl (Fig. 8), consistent with mild, and late-onset, glucose intolerance in these animals. Glucose-induced Ca^2+^ increases were nonetheless significantly delayed in the KO animals (Fig. 10). By contrast, when fed on RC, islets from female βVps13cKO mice displayed a significant enhancement in glucose-stimulated Ca^2+^ increases vs. islets from control littermates (Fig. 11*A*), whereas GSIS was unaltered (and tended to be decreased in response to KCl), as mentioned above. These observations suggest that the Ca^2+^ responsiveness of the secretory machinery to intracellular Ca^2+^ increases may be diminished in female βVps13cKO mice, perhaps reflecting changes in the number of fusion-competent secretory granules, as reported after manipulation of the GWAS gene TCF7L2 (81) or the microRNA miR124 (4) and might suggest a common mode of action of genes affecting T2D risk. Finally, it is possible, given that proinsulin levels were elevated in carriers of risk alleles, that prohormone processing is altered after Vps13c inactivation (67). To investigate this hypothesis, we measured random-fed insulin and proinsulin concentrations in RC-fed mice. No differences were seen in either insulin or proinsulin plasma concentrations, nor was the insulin/proinsulin ratio different between controls and KO mice (Fig. 13).

Changes in glucose tolerance were not apparent after the maintenance of mice (either male of female) on a HFD, suggesting that the phenotype might be rescued by changes in response to high-fat feeding and insulin resistance. One possible explanation might be an increase in the expression of other VPS13 family members, which could be triggered by a HFD, thus compensating for the absence of VPS13C. According to a recent study of mouse islet cell transcriptomes (5), Vps13c mRNA levels are two to three times those of Vps13a, -b, and -d in the β-cell under conditions of normal feeding. Whether changes in the expression of any of these genes occur under the stress of a HFD has yet to be investigated. We note also that a recent eQTL study (9) did not report a significant association with VPS13C (or other genes at this locus) and T2D risk, although whether the latter study was adequately powered to detect small changes is uncertain.

How might VPS13C influence insulin secretion in vivo? Clues might be gleaned by comparisons with other members of the VPS13 family. BLAST analysis of the protein sequences of the four family members indicates that VPS13C is most similar to VPS13A, sharing 41% identity (73) and the two proteins possess several common domains and have similar NH_2_ and COOH termini, indicating that they may have similar functions (73). Both can attach to membranes, although VPS13C has intramolecular duplications vs. VPS13A, which may imply neofunctionalization (i.e., the acquiring of new roles) compared with VPS13A (73). As noted above, a loss of VPS13A (also called chorein) expression leads to the rare neurodegenerative disease chorea-acanthocytosis (ChAc) (53, 71). Symptoms include cognitive dysfunction, hyperkinetic movement disorder, and erythrocyte acanthocytosis (72), leading to significant disability and a reduced life expectancy. Since the discovery of the cause of ChAc, much work has been done to investigate the molecular function of VPS13A. The protein has been localized to endosomal structures in yeast and erythrocytes (14, 28, 64, 70) as well as to the Golgi, and cofractionates with dense-core vesicles in synaptosomes (25, 34). VPS13A is also implicated in a plethora of cellular processes in different settings, including regulation of the actin cytoskeleton (2, 18, 64), protein trafficking (7), membrane morphogenesis (48), autophagy (45), and phagocytosis (59). Cells depleted of VPS13A have decreased levels of PI(4)P and of phosphorylated PI3K (18, 47, 48). Importantly, further evidence for a role for VPS13A in the control of regulated exocytosis was provided recently by Hayashi et al. (25), who demonstrated that VPS13A is localized to neurites in dopaminergic PC12 cells. These findings are thus strongly reminiscent of our findings here of colocalization between VPS13C and insulin in human β-cells (Fig. 12). The role for VPS13A in phosphoinositide (PI) metabolism is a function that is conserved between yeast and human orthologs and a possible mechanism by which VPS13A can function in so many different cellular processes (18, 47–49). If VPS13C were to have similar functions in β-cells as VPS13A, we would hypothesize that the former might be involved in protein trafficking, potentially through the regulation of PI metabolism.

Correct regulation of PI metabolism is essential for efficient insulin secretion from β-cells (79). Indeed, PI(4)P is the main precursor to form PI(4,5)P_2_, which is rapidly turned over to form second messengers required for insulin secretion (68) in a Ca^2+^-dependent manner akin to the release of neurotransmitters from neurons. Interestingly, a distinct role for PI(4)P in signaling from the plasma membrane in the β-cell has been suggested (80), since PI(4)P displayed antisynchronous oscillations compared with PI(4,5)P_2_ when MIN6 β-cells were stimulated with glucose. A direct role in secretion has already been shown in yeast (24), and it is well known that PI(4)P is involved in membrane trafficking between the Golgi and the plasma membrane and other endosomal compartments (75). Hence, VPS13C could function in insulin secretion through regulation of PI metabolism, affecting intracellular insulin trafficking.

Interestingly, new work shows that VPS13C is involved in lipid droplet formation and regulation of galectin-12 and seems to function in adipogenesis (84). The latter findings indicate that VPS13C may play additional roles in T2D in extra-pancreatic tissues.

In conclusion, human islet expression data suggest that variations in the level of expression of VPS13C and C2CD4A in the β-cell may contribute to altered T2D susceptibility in risk allele carriers, at least in females. The relatively mild effects of Vps13c ablation on glucose homeostasis are consistent with the hypothesis that changes in the expression of both genes may contribute to overall risk. Future functional studies will be required to determine the role of C2CD4A in the control of insulin secretion and the possible contribution of indirect mechanisms resulting from changes in the expression of either gene in extrapancreatic tissues.

## GRANTS

G. A. Rutter thanks the Medical Research Council (UK) for Programme grant MR/J0003042/1, the Biotechnology and Biological Sciences Research Council (UK) for a Project grant (BB/J015873/1), the Royal Society for a Wolfson Research Merit Award, and the Wellcome Trust for a Senior Investigator Award (WT098424AIA). T. J. Pullen was a Diabetes research and Wellness Foundation postdoctoral Fellow (SCA/01/F/12). The work leading to this publication has received support from the Innovative Medicines Initiative Joint Undertaking under Grant Agreement no. 155005 (IMIDIA), resources of which are composed of a financial contribution from the European Union's Seventh Framework Programme (FP7/2007-2013) and EFPIA companies' in-kind contribution (G. A. Rutter, P.M.). Additional support was obtained from a Wellcome Trust core grant (075491/Z/04) and from the Advocacy for Neuroacanthocytosis Patients (to A. P. Monaco and A. Velayos-Baeza).

## DISCLOSURES

No conflicts of interest, financial or otherwise, are declared by the author(s).

## AUTHOR CONTRIBUTIONS

Z.B.M., N.F., M.C.C., M.H., P.C., G.M., A.V.-B., A.P.M., L.M., and P.M. performed experiments; Z.B.M., N.F., T.J.P., M.C.C., M.H., P.C., G.M., A.P.M., and G.A.R. analyzed data; Z.B.M., N.F., T.J.P., and G.A.R. interpreted results of experiments; Z.B.M., T.J.P., and M.C.C. prepared figures; N.F., T.J.P., M.C.C., M.H., P.C., G.M., A.V.-B., A.P.M., L.M., and P.M. approved final version of manuscript; A.V.-B. and P.M. edited and revised manuscript; G.A.R. conception and design of research.
